# Understanding the lived experience of pregnancy and birth for survivors of rape and sexual assault

**DOI:** 10.1186/s12884-023-06085-4

**Published:** 2023-11-16

**Authors:** Rebecca Lissmann, Michelle Lokot, Cicely Marston

**Affiliations:** 1 Queen Mary University of London, Yvonne Carter Building, 58 Turner Street, London, E1 2AB England; 2 London School of Hygiene and Tropical Medicine, 15-17 Tavistock Place, London, WC1H 9SH England

**Keywords:** Pregnancy, Birth, Survivor, Rape, Sexual Assault, Qualitative

## Abstract

**Background:**

One in five women in the UK are survivors of rape and sexual assault, and four in five women will give birth. This implies that a substantial number of women experience rape and sexual assault before pregnancy. We highlight and explore the voices and lived experiences of survivors during pregnancy and birth, to better understand the relationship between sexual violence, biomedicine, and pregnancy and to inform maternity care practice.

**Methods:**

This qualitative research took an intersectional feminist approach. We conducted in-depth individual interviews in England with fourteen women who self-identified as survivors of rape or sexual assault, and who had experienced pregnancy and birth after the assault. We conducted open line-by-line coding of the interview transcripts, and identified key themes and sub-themes inductively.

**Results:**

Three themes help summarise the narratives: control, safety and trauma. Maintaining a sense of control was important to survivors but they often reported objectification by healthcare staff and lack of consent or choice about healthcare decisions. Participants’ preferences for giving birth were often motivated by their desire to feel in control and avoid triggering traumatic memories of the sexual assault. Survivors felt safer when they trusted staff. Many participants said it was important for staff to know they were survivors but none were asked about this. Pregnancy and birth experiences were triggering when they mirrored the assault, for instance if the woman was prevented from moving. Many of our participants reported having unmet mental health care needs before, during or after pregnancy.

**Conclusions:**

Survivors of sexual violence have specific maternity care needs. For our participants, these needs were often not met, leading to negative or traumatic experiences of pregnancy and birth. Systemic biases and poor birth experience jeopardise both psychological and physical safety. Funding for maternity and mental health services must be improved, so that they meet minimum staffing and care standards. Maternity services should urgently introduce trauma-informed models of care.

## Background

This research focuses on the experience of pregnancy and birth for survivors of rape and sexual assault (RSA). RSA is common: In the UK, 20% of women have experienced sexual assault [1]. In a sample of 22,419 UK women, 99.3% reported that they had been subject to sexual harassment, sexual assault or rape [2]. In the UK, 80% of women turning 45 years old in 2019 had given birth [3]. The overlap of these experiences is likely to affect a high proportion of the UK population. Existing literature suggests that RSA can negatively affect women’s experience of pregnancy and birth [4–11]. Understanding survivors is essential to providing maternity care that meets their needs.

Rape is legally defined as a person intentionally penetrating another’s vagina, anus or mouth with a penis, without consent; sexual assault is defined as intentional sexual touching without consent [12]. RSA often has a significant impact on the survivor. Physical consequences may include injury, sexually transmitted infection, or pregnancy [13]. Psychological diagnoses following RSA include post- traumatic stress disorder, anxiety, depression, and patterns of self-blame and low self-esteem [14]. Social impacts stem primarily from victim blaming and secondary victimisation [15] but can extend further, for example, by reducing survivors’ ability to work and harming their economic well-being [16].

The concept of ‘rape culture’ frames rape as a violent crime and tool for maintaining patriarchy i.e. an overarching system of power founded on men’s dominance, women’s subordination and ‘men’s ownership and control of women’s reproductive powers’ [17, 18], rather than as a sex crime motivated by pleasure [19]. Patriarchy intersects with other power hierarchies, including racism, to sustain men’s use of force as a means of exercising power [20].

Young’s [21] concept of pregnant embodiment suggests that the discourse on pregnancy within healthcare settings omits subjectivity as it does not take adequate account of that individual’s personhood or perspective. Rothman [22] similarly reasons that women are not ‘flowerpots’ in which babies are planted, but social beings with a social relationship to their pregnancy and the healthcare professionals involved in their care. Pregnant women may be reduced to vessels from which information about the foetus must be obtained, through machines or examination [21]. Such instrumentalisation of bodies is exemplified by one study on the emotion management of women with medically high-risk pregnancies where, for example, one woman was referred to as a ‘great incubator’ [23]. Ideas about danger can be used to re-establish positions of power, with risks to foetuses ‘deployed to govern women’s behaviours and bodies’ [24].

Experiences of pregnancy and birth can be negative for many individuals. In an Australian study of 933 women, 866 were followed up to 6 weeks postpartum; 45.5% of this group reported that they had experienced a threat to themselves or their baby, and had experienced fear, hopelessness or horror during birth [25]. In a smaller US survey, 34% of 103 women reported their experience of giving birth was traumatic [26]. In an international survey of 943 women, 748 responded to a free text question ‘describe the birth trauma and what you found traumatising’; themes included ‘prioritising the care provider’s agenda’; ‘disregarding embodied knowledge’; ‘lies and threats’; and ‘violation’. Participants described how providers sometimes implied the wellbeing of the baby was at stake in order to coerce them into complying with different procedures [27]. Other research shows that women’s recollections of birth are related more to whether they were able to choose what happened than to whether the birth would be considered medically complicated [28]. Although being an RSA survivor was not a criterion for participation in these broader studies of birth experiences, when pregnant women are research participants, many report experiencing traumatic events prior to birth (91% of 933 participants in one study for instance) [25], or that actions of healthcare staff during birth triggered memories of RSA [27]. Trauma, sexual harassment and RSA are such common experiences for women that it is imperative to take them into account when studying experiences of pregnancy and birth.

Despite the importance of the topic, there have been few qualitative studies exploring the experience of pregnancy and birth for survivors of RSA. In one UK study, nine in-depth interviews were conducted with women who had lived through childhood sexual abuse (CSA); it explored their experience of pregnancy and birth, and included themes on loss of control, pain, encounters with strangers, triggers and re-enactment, or mirroring, of abuse through the way in which intimate procedures were conducted [4]. Most recently, it has been proposed that strategies developed to survive CSA can be (re)activated during pregnancy and birth [5]. This suggestion is supported by an earlier study in Florida, featuring interviews with seven women who had experienced CSA. It showed that sensory memories of abuse can be ‘tripped’ during birth, causing remembering or reactions, such as panic, without women recognising at the time that their reaction was due to prior abuse [6]. In a larger study of 85 German CSA survivors, 41% reported experiencing intrusive memories of their trauma during birth [7]. A study in Connecticut recruited eight survivors (six of CSA, one of rape and one of both); many themes were explored, all connected by survivors’ overwhelming desire for control [8]. A further study in the United States of America (US) conducted narrative interviews with 15 women with a history of CSA, finding that they wanted maternity care providers to pro-actively address their trauma-related needs [9]. A Norwegian study interviewed 10 women who were raped in adulthood, and found that trauma may be reactivated during birth, with all participants describing ‘being back in the rape’ [10], much like the UK study on CSA. In the largest US study, interviews with 20 survivors of both CSA and rape in adulthood were compared with those of 10 women without a history of sexual trauma. The study found that survivors wanted to be offered female providers, and for healthcare professionals to avoid triggering language; survivors wished to be supported in controlling whether their their body was exposed and who could enter their labour room [11].

The experience of pregnancy and birth, and listening to pregnant people’s lived experiences and voices, is not just desirable, but essential to providing safety within maternity care - in the UK and worldwide. The 2017–2019 UK and Ireland national inquiry into maternal deaths and mortality (known as MBBRACE) found that Black women are more than four times and Asian women almost twice as likely to die in the perinatal period than white women. The inquiry emphasised that addressing intersecting structural biases is fundamental to the prevention of maternal mortality [29]. The subsequent Birthrights inquiry into racial injustice found that racism infringes basic human rights in maternity care; the inquiry found that women of colour experienced a lack of physical and psychological safety, were ignored or disbelieved and dehumanised; lacked choice and sometimes experienced coercion [30]. National inquiries into UK National Health Service (NHS) failings, including the Francis report [31], Morecambe Bay [32], Cumberlege report [33] and Ockenden report [34], all identified patterns where individuals who raised concerns were not listened to, with consequences for physical safety. Cumberlege summarised by stating that patient experience ‘must no longer be considered anecdotal and weighted least in the hierarchy of evidence based medicine’ [33].

The duty to uphold dignity is encoded in professional standards for doctors, nurses and midwives [35, 36]. In Canada, Jacobson found that violation of social dignity is more likely in settings with asymmetrical relations between and within groups [37]. Hierarchies in healthcare relate not only to qualifications and experience but also to social hierarchies such as race, gender and class [38, 39]. This effect is greater in strained and resource poor clinical settings [37]. In 2021, the Health and Social Care Committee estimated that £200–350 million extra funding per annum is required to tackle shortages of midwives, obstetricians and other maternity staff in the NHS [40].

Studies that have been conducted in the UK focussed on survivors of CSA and their later pregnancy experiences. There is a lack of research on pregnancy and birth experiences in the NHS for rape and sexual assault survivors. In this study we focused on the experiences of women who survived RSA without becoming pregnant due to the assault, then later in life experienced pregnancy and birth. We aimed to highlight and explore the voices and lived experiences of survivors during pregnancy and birth, to inform better maternity care services and practice, and to better understand the relationship between violence, biomedicine, and pregnancy.

## Methods

### Study setting

The study was undertaken in England during June and July 2021. Interviews took place online, in participants homes or, in a few instances, at a rape crisis centre in North England. Participants were located across England.

### Study Design

This qualitative research took an intersectional feminist approach. We conducted in-depth interviews with survivors of RSA, aiming to explore their lived experience of pregnancy and birth. Feminist principles included flattening hierarchies, an ethic of care, reciprocity, reflexivity and relational interviewing [41–43]. An interpretivist to constructionist approach informed the use of qualitative in-depth interviewing.

### Sampling technique and sample size

Twelve rape crisis centres in the East of England (4), South East (2), East Midlands (1), West Midlands (2) and North West (3) supported the research. Other supporting organisations included ‘The British Pregnancy Advisory Service Centre for Reproductive Research and Communication’, Decolonising Contraception and Birthrights. Our advert contained a research description and contact details. Supporting organisations shared the advert via their networks, social media channels and newsletters. We shared the advert in our networks. Participants volunteered to take part by contacting RL, who screened them against inclusion criteria: over 18 years old, living in the UK, self-identifying as survivors of either rape or sexual assault at any age, experienced pregnancy and birth after the assault. Participants of any gender, ethnicity, socio-economic grouping or location within the UK were included. Interested participants were sent the participant information sheet. Most were available for a phone call to discuss the research and establish a relationship. Decisions regarding the interview location, time, date and length were the participants’ choice. Participants digitally signed the consent form prior to interviews. Participants were encouraged to contact RL if they had further questions. All participants were compensated with a £5 voucher of their choice. Fourteen women participated in the research. All had given birth in the UK, except one person who had given birth elsewhere.

### Data Collection Procedure

Fourteen people agreed to participate in the research following the initial recruitment effort. Ten interviews were held in person and four were held via Zoom video call with end-to-end encryption enabled. Interviews were held via Zoom either at the participant’s request or due to travel limitations. Eight of the in-person interviews were held in the participant’s home, and two were held at a rape crisis centre. Only the participant and RL were present for interviews.

RL conducted and audio-recorded all interviews. Interviews were 60 to 135 minutes long, with most lasting approximately 90 minutes. The interviews explored themes including pregnant selfhood, narratives of survival, interactions with clinicians and networks of support. Interviews began with broad, less sensitive questions such as ‘Can you tell me a little about your life at the moment?’Follow-up questions explored topics raised by participants. We present below the responses to these questions which appeared across multiple interviews.

### Data analysis

Data were anonymised. Participant information and data were stored separately and securely. RL transcribed ten interviews and an external agency transcribed four of them. RL checked each transcript for accuracy and used NVivo12 to code transcripts iteratively during data collection. RL familiarised herself with the data by transcribing and reading through transcripts, reviewing field notes and discussing data with the research team. Open line-by-line coding formed a series of codes which ranged from descriptive to conceptual. Second level coding then brought together open codes. This approach allowed themes to be derived inductively. Themes were reviewed and discussed by RL and ML to identify three key themes. Two to three connecting subthemes were developed under each key theme. Feminist epistemology and intersectional feminism informed analysis, exploring how gender interacts with power structures such as neoliberalism and racism, and rooting knowledge in the lived experience of participants [41].

### Research Team and Reflexivity

This paper was part of RL’s Masters dissertation, supervised by ML and CM. RL is a junior doctor training in obstetrics and gynaecology. ML is an Assistant Professor and CM a Professor at the London School of Hygiene and Tropical Medicine (LSHTM). RL used reflexive approaches including journaling, developing field notes, analysing power dynamics with participants during interviews and seeking to reduce hierarchies, and discussing reflections on power and positionality with others, including the co-authors. She approached interviews as a relational process, where accounts were co-created. She aimed to build reciprocity, trust and solidarity with participants, irrespective of whether interviews were in person or online, including answering interviewees’ personal questions.

### Trustworthiness

To maximise usefulness of the study, we took steps to ensure different voices were incorporated and used rigorous analytical methods to fairly represent the interview data. In addition, the themes we identified align with and expand on existing evidence from other sources. For these reasons, we would anticipate similar findings would be yielded from other, similar, settings if our study were replicated elsewhere in the UK and possibly more widely. In this sense the research is credible, confirmable and transferable.

### Ethical consideration

This study was approved by the LSHTM ethics committee (reference 25,856). Throughout the research, we prioritised participants’ wellbeing. RL undertook online training on working with sexual assault survivors, and developed a protocol in advance to handle any cases of participant distress.

## Results

Participants were from mixed socioeconomic backgrounds. All reported current or past heterosexual relationships and one spoke about her lived experience as a Black woman. Participants were not explicitly asked to define their gender identity, however nine referred to themselves as women. It is possible that some participants may identify in other ways. Of the fourteen participants, five were survivors of rape and sexual assault during childhood, seven during adulthood, and the final two described RSA during both childhood and adulthood.

Three linked themes help summarise the narratives: (1) Control – including sub-themes of objectification and birth preferences; (2) Safety, with sub-themes including trust and disclosure; and (3) Trauma, including sub-themes of triggers, mental health and multiple traumas.

### Control

Control and consent were described by participants as important in their experiences of pregnancy and birth, linked to the concept of lost bodily autonomy. Survivors expressed an understanding that birth carries clinical risk and is an unpredictable process. However, they wished to exert as much control as possible: *‘I know that birth is uncontrollable, but what I can control of it, I want to.’* Control and consent came up frequently:

*[If] you’ve been in a situation where you’re so vulnerable, and you’ve lost control of your body and your rights to your body and your rights to say no, or even, in my case, my rights to wear pyjamas to bed. To be able to have the control to go, I’d like this to not happen, is really important.*

Only two participants reported that they gave informed consent throughout their care. Five survivors described experiences where no attempt was made to gain consent. For example: *‘And there was just suddenly all these people, and all these hands, and nobody was really saying ‘I’m gonna do this, I’m gonna do that’ it was all just kind of like that.’*

Participants often said that they had been told they would be examined, rather than asked whether they consented to be examined: *‘And they said, ‘The student’s going to examine you.’ And that’s when I thought, well I put in my notes that I don’t want her to. But by that point I felt a bit, sort of… bit frightened, really. And so, she examined me…’*.

Participants were not always listened to. One participant was a Black woman and had presented in pain repeatedly before she was investigated and treated for appendicitis during pregnancy. She identified the role of racism:

*[T]here’s just been a whole load of, I guess, like fresh awareness around just Black women, women of colour not being taken seriously. And I just had never, probably ignorantly, just known that that was a thing. Just had not realised that… when we do present in pain, people were just like… either you’re not in pain, or you’re not in as much pain as you think you are.*

In accounts where a discussion did occur, some described a sense of false choice or coercion. Participants explained how vaginal examinations were presented as compulsory in order to access other elements of care:

*And the midwife said to me, she was like, ‘You don’t have to have an internal exam. But the doctors in theatre, male doctors in theatre, won’t admit you unless I know how many centimetres you’re dilated. So it’s a case of you don’t have to have this exam but if you don’t, they’re not going to take you down for the C-section’. So it’s like, I do have to have it then don’t I?*

Participants shared how the baby’s needs may be prioritised above the pregnant person’s, reducing women’s control over decisions. For instance one woman who declined a Caesarean-section earlier in labour said she felt she *‘didn’t have a choice anymore’* when she was told her baby was distressed.

### Objectification

Many participants felt objectified by healthcare staff, commonly using the words *‘object’* and *‘vessel’* to describe their experiences. Three different survivors spoke about being made to feel *‘like a piece of meat in butcher shop’*, *‘like a greenhouse’*, and as if they were not *‘in the room’*. Another reported that she felt *‘positioned as obstructive’* and *‘…just this annoying thing that surrounded the important part’*. In some cases, the objectification was more subtle:

*[W]hen she [baby] was delivered, he [partner] was running round the operating theatre like she was the FA cup, that’s how I thought of it. And they were saying, show her the baby, show the mum the baby. And in the end the midwife had to grab her and pull her off him and put her next to me so I could see her…*.

In another account, a participant was becoming increasingly unwell. She had *‘begged’* for a Caesarean section over several days, when a new consultant arrived:

*[H]e said to me, I’m not being funny and I don’t want you to take this the wrong way, but your baby’s not my patient, you’re my patient. And at this point she’s a parasite and she’s hurting you, so we need to get her out. And I’d never met him before but I trusted him from that moment, straight away.*

### Birth preferences

Whilst control was important to all participants, they held different views on what kind of birth would make them feel most in control. Four survivors expressed a preference for Caesarean section. Reasons for this included hoping to avoid vaginal examinations and tearing, to know what would happen in advance, to give birth calmly and to avoid an emergency or an influx of people into their birthing space. One woman’s first birth was vaginal and her subsequent birth was by Caesarean section:

*And the funny thing is, I did feel more in control in a way with the Caesarean. Because they explain everything to you, it’s all very predictable, isn’t it, I suppose in a way that birth isn’t. So, you know, they say, ‘We’re going to do this now, and this will happen’. And they do it and it happens. And- not like birth, where you go in and it’s like… going through a minefield with your eyes closed.*

By contrast, six survivors spoke about the importance of a non-medicalised birth. They valued, for example, the opportunity to give birth in a room with just a few people in it, without medication that might render them immobile, and without the sense of objectification caused by having things done to them by healthcare staff. The participant who gave birth outside the UK echoed comments about control made by other participants who had given birth in the UK, when she said:

*[I]f you just think of a Caesarean section, you can’t- you can’t do or be anything that you are, because essentially, they are performing surgery on you. And they dictate how your body should lie on the table, who is going to perform what on your body. You have no say. That’s what I feel like, for me, is like the handing over of my body. This erm… complete loss of control, essentially.*

Survivors described holding these differing birth preferences for similar reasons: to maintain a sense of control, or to avoid the birthing process from triggering memories of the assault. Many participants described strongly advocating for their birth preferences: they described ‘*negotiating’*, ‘*manoeuvring’* and ‘*fight[ing]’*, or using their social status, education, or skills. Others employed internal coping mechanisms: *‘I didn’t really connect with my pregnancies. I wasn’t- I was quite good at being pregnant because it didn’t really… there was a bump. And then there was a baby. There was no baby before she was there and she was on me.’.*

### Safety

Participants said they wanted to give birth safely. However, they spoke about safety holistically rather than only in relation to physical risk, referring also to the environment and the people around them. One survivor contextualised the importance of safety:

*Like, if I’m around people that get me and accept me, then I feel safer… I don’t have to constantly explain myself or be like, oh, that’s not what I meant. Or, like, so it means that I can be calm. And I guess in a kind of cavewoman sense, if I’m not thinking about all those things, I’m gonna see the sabre-tooth.*

She later explained that the sabre-tooth symbolised danger and trauma. She wanted to reduce her mental load so that she would spot danger approaching. Other participants also mentioned primal or intuitive needs when discussing safety.

### Trust

Survivors felt safer if they trusted the maternity staff looking after them. Only three participants reported seeing the same healthcare provider repeatedly during their antenatal care. Survivors described developing trust in a doctor or midwife when they gave consistent, personalised information, shared the same lived experiences, for example as a Black woman, or showed an interest in knowing them as an individual, by reading their notes, listening, or using their name:

*I’m talking in a kind of wishful thinking way, because I appreciate that it’s- it would be impossible really to do it. But I think I would’ve felt safer, more trusting of my doctor if he’d known my name, or if he was even able to give the illusion that he knew my name. And- and feeling like he’s genuinely interested… in what I want from- from my pregnancy and from my birth. He’s genuinely interested in my wellbeing.*

Almost every survivor described wanting to be seen as a whole person and ideally to interact with a healthcare professional who reciprocated by sharing something of themselves. This was challenging in the context of systematised healthcare and objectification: *‘It makes it quite difficult to feel like a person, rather than kind of like a number in the system, to really feel seen.’*

Some survivors felt that maternity staff needed to know their status as survivors in order to care for them properly. One participant described that no one asked whether she was a survivor, despite signs. She felt they ignored her because talking about sexual assault was too difficult. Yet, she saw her status as a survivor as an essential part of her history: *‘[If] I don’t tell somebody that, then they don’t know me.’* When another survivor expressed anxiety about vaginal examinations, the midwife responded: *‘well, put it this way, if you’ve managed to get a penis in there to get yourself pregnant, you’ll be able to have this examination done.’* This was not a safe space to disclose.

### Disclosure

Nine participants realised their sexual assault was relevant to pregnancy and birth either before or early on in their pregnancy. The remaining five came to realise this in retrospect. None of the participants were asked whether they were survivors and the main reason for choosing not to disclose was that the healthcare provider did not ask. Five people disclosed during their pregnancy or labour. Of this group, three disclosed in the context of explaining their birth preferences for either a Caesarean section or midwife led care. Despite it being documented in their notes, all three reported having to disclose repeatedly in order to explain their preferences. One described this vividly:

*[I]t just felt like I was pulling the top layer of my skin off over and over again and leaving my nerves exposed. And nobody ever treated it, they just kept pulling my skin off, and my nerves were just exposed. So every time I went in there and just go, well, this happened, and really quickly tell them this really massive thing.*

Participants reported that reactions to disclosure reflected discomfort, inadequate knowledge of how to respond and sometimes disbelief. One was asked *‘This was ten years ago, why are you bothered?’.*

Another participant disclosed but also described not having insight into the relevance of her sexual assault at the time. During her second labour, the midwife explored what had been difficult about her first birth, and she disclosed within this broader conversation. This survivor later connected her disclosure with her midwife’s decisions to be *‘hands off’* and avoid triggering her. She went on to share her positive birth experience:

*I was like, filled with romance about how wonderful birth is and how amazing I am because ‘Look what I’ve just made’. And I was so proud of myself that I decided I was going to do it again… Third time round I was a surrogate…*.

### Trauma

Events during pregnancy and birth may cause retraumatisation by triggering past trauma from rape or sexual assault. One participant highlighted that her birth was traumatic because the behaviour of maternity staff mirrored that of her abuser, rather than because of medical complications:

*It was just traumatic- it was just the trapped- it was people sort of, you know grabbing onto your thighs and pushing your legs and doing things with your body that I’ve obviously experienced before under different circumstances and every time it happened just another image in your mind. So you just lay there, like you’re going through it all over again.*

Two participants said that they wanted more children but had chosen not to. They said they could not risk further trauma by going through another pregnancy and birth.

### Triggers

Triggers, both anticipated and experienced, were linked to survivors’ sexual assaults and other lived experience as women. They influenced their birth plans:

*I don’t want men involved in my antenatal care or labour care… when I’m in a space where I’m going to feel vulnerable anyway, I just don’t want to hear men’s voices in my ear, even if they’re nice people.*

Seven survivors found men triggering. For two it was important that men were not involved in their care. Some survivors saw the potential for harm without intent. However, another recounted one vaginal examination, which she named as sexual assault: *‘He was doing an internal examination and I told him, stop. And he didn’t. And I told him stop. And he didn’t. And I shouted at him to stop. And he didn’t. And he only stopped when my husband said stop…’*.

### Mental Health

Four participants felt anxiety during pregnancy. After birth, two reported low mood, and a further two had symptoms of psychosis. Many described symptoms associated with post-traumatic stress disorder (PTSD) during their pregnancy or birth, which included flashbacks, nightmares, a sense of being ‘in’ the trauma, avoidance of trauma related stimuli, or finding their memories muddled between their birth and sexual assault. Three participants did not describe any mental health symptoms; some said they had processed their trauma before becoming pregnant.

Only one survivor saw a mental health specialist ‘pre-crisis’ under the NHS; she was seen by a nurse who expressed disbelief that she was a survivor: *‘…she was amazed that I’d been able to have sex to get pregnant as a survivor of rape… She just didn’t believe me that- that I could possibly have been raped and then have a healthy sex life thirteen years later.’*

Another participant took an overdose because her mental health declined during pregnancy and she could not cope with her symptoms. She was taken to a psychiatric hospital:

*[N]o one had said but it was PTSD… constant flashback[s]… nightmare[s] all the time… And then when you’re pregnant, all the thing[s] they have to do, which… triggers some of the flash back- a[s] well. So [pause] yeah, that was really hard. Even though I really wanted this baby.*

One woman’s abuse began shortly after she gave birth to her first child. In her following pregnancy the delivery suite was triggering. She was referred to the perinatal mental health team early in her second pregnancy, but was not seen until 8 months post-partum. In the absence of the mental health team, her midwife provided significant psychological support, and offered her the option to give birth elsewhere. By contrast, another woman’s health visitor missed the opportunity to support her:

*So I- I- told her that I’m not feeling too well, I’m- sometimes I think I might… I’m gonna die or I had really negative thoughts. They said, ‘Okay, just wait a little bit. Maybe it’s just your hormones, you just gave birth’. And I think after that, I started to think- and I was really worried that they would take my [baby] away from me. And after that, even if I didn’t feel well I just said, ‘I’m alright.’*

She was not able to access further support until her child was two years old. As she waited for mental health services, the same participant described fear that her leave to remain was under threat: *‘…we have got also Brexit during all these things. So I was afraid that- oh, all these things come back to me because I’m not English.’*

Several survivors felt that mental health services did not meaningfully engage in treating trauma caused by sexual assault. Instead, they were referred to rape crisis centres, which are charities with long waiting times. They wanted a service that connected sexual assault, maternity and perinatal mental health care.

### Multiple traumas

Participants described layers of trauma experienced during their lives. Sexual harassment was common: on the morning of the interview, one survivor described a call from a private number with *‘somebody wanking down the phone’*, another mentioned avoiding walking at night. Some had lived through other trauma, including child abuse, being in the care system, domestic violence, police brutality, abduction and miscarriage. They navigated challenges, such as moving house, family disputes, illness or deaths, and financial hardship. One participant summarised: *‘I’ve never had a period of time in my life before my children came along where everything was okay. For any length of time longer than just getting through the day.’*

For one survivor, her sense of vulnerability and the lack of consent during labour were consistent with her sexual assault, as both a child and adult, up to that point: *‘…it was just- and similar to, you know, what- the assault and things. It was- it was like you’re there for anybody’*. Among our participants there was no specific difference evident between those who survived sexual assault as children or adults. However, only people who survived childhood sexual abuse expressed a sense that the trauma they experienced during birth fit in to how things had always been for them. Not all of the sub-group described feeling that way.

## Discussion

This study shows how neglecting to take women’s experiences of rape and sexual assault into account during maternity care can lead to negative or traumatic experiences of pregnancy and birth. Women are confronted by the consequences of gender-based violence throughout the life course, including during pregnancy and childbirth. Participants’ accounts depict women’s positioning within a health system that often reinforces patriarchal structures and norms. Survivors carry the impact of past traumas into pregnancy and birth and prioritise retaining a sense of control and feeling safe. These priorities are often not elicited, explored, or addressed by those responsible for their care.

Informed consent is a professional standard, ensuring women retain bodily autonomy and dignity [35, 36]. Previous studies have found that maintaining a sense of choice and control is important to pregnant people generally [28] and is particularly important to survivors of rape and sexual assault [4, 8, 11]. This study suggests that healthcare professionals did not always ask for informed consent or offer adequate choice. Our results are also supported by prior studies indicating that when staff recommend interventions as ‘safest’, questioning the need for the intervention can be perceived as transgressive. Women can be ‘coerced’ into complying with procedures as part of prioritising the baby’s survival [21, 27]. This echoes literature on pregnant women being rendered vessels or ‘flowerpots’ [22] within medical contexts. Their own identities may be eroded by the focus on their role in relation to their baby, as future mothers [21, 22]. An inquiry into racial injustice in UK maternity care identified themes overlapping with this research: dehumanisation, lack of choice, consent and coercion, and a lack of physical and psychological safety [30].

Being seen as a person with their own identity was important both to the process of consent and in building safety through trusting connections with maternity care staff. Continuity of care can facilitate trusting, reciprocal relationships [44]. Although NHS England launched smaller ‘continuity of carer’ teams, the Ockenden report recommended their suspension nationally because this model ringfences midwives to care for certain pregnant people and cannot be safely implemented where total staffing in inadequate. [34, 45]. For our participants, trust was also built when staff demonstrated an interest in getting to know them as people. For some, their status as survivors was an important part of being known. Existing literature has highlighted how participants want staff to address their needs as survivors [9]. However, none of our participants reported being asked about their history of RSA, and those who disclosed sometimes encountered disbelief and ignorance. Important opportunities to identify and care for survivors were missed.

In line with previous literature [4, 6, 7, 10], our study shows that survivors with trauma from RSA face the possibility of new trauma or retraumatisation during pregnancy and birth. Psychological trauma during birth can lead to PTSD [25]. Although NHS England aims to provide perinatal mental health care up to 24 months postpartum, there are long waiting times for appointments [45]. Similarly, commitments to ‘lifetime NHS mental health care for sexual assault victims’ [46], are not always borne out in practice and survivors’ accounts here indicate that accessible, integrated care remains an aspiration.

The 2017-2019 national MBBRACE report stated that addressing ‘structural biases affecting women’s care on the basis of their pregnancy or the potential to become pregnant is fundamental to preventing maternal mortality…’ [29]. These structural biases concerning, for example, gender and race, permit a healthcare system where some pregnant people are not asked for consent, feel unsafe and are re-traumatised. The same patriarchy that permits RSA also permits this healthcare system. Multiple inquiries have noted that the failure to respond to patients’ and pregnant people’s concerns is a key contributor to failings in the healthcare system [31–34]. Both safety and experience are important and interconnected. The participants in this research did not solely hope to survive pregnancy and birth; they had more holistic hopes and needs, which were often not met. Crucial elements of their care, particularly control, trust, and managing trauma, were neglected.

In this study we were not able to assess whether there are differences between diverse groups of pregnant people. There may be important additional issues to consider for different marginalised groups, such as disabled people. Factors such as race, sexuality and class interact with cultural differences, and social phenomena and so may produce additional findings.

## Conclusions

A substantial proportion of women who become pregnant and give birth in the NHS will be survivors of rape and sexual assault. Healthcare professionals must take this into account by ensuring they obtain informed consent, provide choice and enact simple changes to build trust, for example by using people’s names rather than generic terms such as ‘mum’. Maternity care leads should ensure teams are trained on trauma and RSA, as part of working towards a trauma-informed model of care [47], so that staff have the knowledge and skills to meet survivors’ needs. Maternity and mental health services must be funded adequately to ensure they meet basic standards regarding staffing and waiting times [34, 40]. This will enable healthcare staff to spend more time with each pregnant person, and make interventions such as trauma therapy available prior to birth for those who disclose during pregnancy.

Participants in this research had specific maternity care needs as survivors. The current widespread failure to recognise the prevalence and impact of rape and sexual assault produces a healthcare system which does not meet survivors’ needs, risking their physical and mental health.

## Data Availability

The datasets generated and analysed during the current study are not publicly available due to the sensitive nature of interviews and possibility of compromising privacy.

## Abbreviations

RSA: Rape and sexual assault
NHS: National Health Service
PTSD: Post traumatic stress disorder
